# The formation of multi-destination image: A study of China’s Greater Bay Area

**DOI:** 10.3389/fpsyg.2022.975025

**Published:** 2022-08-05

**Authors:** Xialei Duan, Ivan Ka Wai Lai

**Affiliations:** Faculty of International Tourism and Management, City University of Macau, Macao, Macao SAR, China

**Keywords:** destination image, structural equation modeling, higher-order construct, regional tourism, Greater Bay Area

## Abstract

Many governments promote the concept of multi-destination tourism to attract foreign visitors to stay longer in a region. This study constructs a higher-order multi-destination image model to examine how the unique cognitive images of Hong Kong, Macau, and Guangzhou collectively constitute the overall cognitive image of China’s Greater Bay Area (GBA). Then, it further examines how this overall cognitive image builds affective, overall, and conative images of the GBA. The results of an online survey of non-Chinese tourists from Guinea, Japan, New Zealand, United Kingdom, and United States show that cognitive images of three cities in the GBA take different weighting in constructing the overall cognitive image of the GBA. The overall cognitive destination image significantly influences the formation of the affective, overall, and conative images of the GBA region. For constructing the conative image, the affective image shows the greatest impact, overall cognitive image follows; the impact of the overall image is less. This study proposes theoretical implications for future regional tourism studies. Practical recommendations are also proposed.

## Introduction

It is important for tourism destinations to keep tourists longer to increase their spending due to tourism’s economic benefits (Khan et al., 1995; Crouch and Ritchie, 1999; Kozak et al., 2008; Jacobsen et al., 2018). To achieve this goal, destination governments have tried to provide a diverse range of tourism products as well as to group several tourism cities into clusters to stimulate regional economic growth (Ferreira and Estevao, 2009; Yang, 2012; Jacobsen et al., 2018). Regional collaborations, which involve co-opetition, co-branding, and joint promotion, have become a trend in tourism development (Cai, 2002; Gooroochurn and Hanley, 2005; Kirillova et al., 2020; Park and Song, 2021). China has recently promoted the concept of the Greater Bay Area (GBA) concept, which is referred to as the Guangdong-Hong Kong-Macao GBA. It is an adjacent metropolitan area comprised of nine cities and two administrative regions in southern China, including core cities such as Hong Kong, Macau, and Guangzhou. The government promotes multi-destination tourism to attract foreign visitors to stay longer in the GBA. Most previous studies of GBA (Kirillova et al., 2020; Park and Song, 2021) were focused on internal perceptions. Thus, it is necessary to determine how overseas tourists could be encouraged to take multi-destination travel to extend their stay. This study intends to fill the research gap by encompassing perceptions of the international market.

In tourism research, a solid and positive destination image is essential to attract tourists to the place (Tasci and Gartner, 2007). Moreover, it also positively influences tourist satisfaction and tourist trust (Jebbouri et al., 2022). From the residents’ perspective, the destination image is also significantly related to the attitude toward tourism development and pro-tourism behavior (Shen et al., 2019). Previous studies have successfully demonstrated destination image models containing different elements (cognitive, affective, overall, and conative image) under different research settings (Agapito et al., 2013; Hernández-Mogollón et al., 2018; Yin et al., 2020). However, these models may not be simply applied to multi-destination tourism because each destination city has unique characteristics that constitute its cognitive image. Each cognitive image of the city may influence the overall image of a multi-destination tourism region. Moreover, a successful multi-destination image may not be a simple aggregation of the image of an individual city. The mechanism for forming a multi-destination image remains unknown. In this regard, if regions like GBA hope to establish a multi-destination image to boost regional tourism, it is necessary to investigate how a multi-destination image is generated.

Nemours research has stated that it is essential for regional destinations to build a successful brand to attract tourists, such by promoting co-operative branding (Cai, 2002; Ruiz-Real et al., 2020), highlighting brand personality (Murphy et al., 2007a,b), lifting brand equity (Gomez et al., 2015). However, branding a destination is largely associated with its image(Blichfeldt, 2005; Tasci and Kozak, 2006; Hosany et al., 2007); we need to investigate how the image is formed in a multi-destination region. This study proposes constructing a multi-destination image model to discover how it functions within the context of regional tourism development. This study selects the grouping of Hong Kong, Macau, and Guangzhou (the capital city of Guangdong Province) to represent multi-destination tourism in the GBA. The selection of these three cities is based on their popularity among international tourists. According to previous research, these three cities are the major tourist destinations for international tourists, especially Hong Kong (Kirillova et al., 2020; Luo and Lam, 2020; Leandro, 2021; Yu, 2021). In other cities in the GBA region, the number of foreign tourists is relatively small, so it is difficult to find foreign tourists’ impressions of their destination. Tourism experts have been suggesting the construction of a GBA tourism zone. As part of a regional plan to attract more tourists, Hong Kong, Macau, and Guangdong province are planning to work together to extend visitors’ average stays in the city to 5 days, starting with packages that will extend visitors’ stay in the GBA region (Tsang, 2018). Guangzhou, as the capital city of Guangdong province, plays a leading role in tourism co-operation. There are already tour packages including Hong Kong, Macau, and Guangzhou on the market (HKTB, 2022). Thus, it is reasonable to choose Hong Kong, Macau, and Guangzhou as our study sites in the GBA region. The study contributes a research framework that provides researchers with better use of multi-destination tourism research. This model explains how the cognitive images of these three cities (Hong Kong, Macau, and Guangzhou) and the affective image of multi-destination tourism formulate an overall image and conative image of multi-destination tourism in the GBA. Secondly, this study contributes to regional tourism research by exhibiting how to de-border tourists’ perceptions of individual cognitive images of cities and then re-border their attributes toward a whole conative image of a tourism region. In addition, this study shows how to evaluate the multi-destination cognitive images by constructing a three-level reflective-reflective higher-order model. Finally, practical recommendations were provided for governments, tourism marketers, and travel agencies.

## Literature review

### Regional image

Destination image study has been popular for decades. It has been widely accepted that there are two major categories of destination components: cognitive image and affective image (Gartner, 1994; Baloglu and McCleary, 1999; Gallarza et al., 2002). Cognitive image refers to a person’s beliefs and knowledge about a destination and its attributes incorporated into a mental representation of the destination (Baloglu and McCleary, 1999; Fjelldal et al., 2021). Affective image shows an individual’s feelings and emotional responses toward a destination (Baloglu and Brinberg, 1997). In addition, scholars have agreed that every place has an overall image, which refers to people’s general impressions of a destination (Baloglu and McCleary, 1999; Yin et al., 2020). Finally, a conative image is akin to action or behavior (Pike and Ryan, 2004; Casali et al., 2021).

Spatial boundaries significantly affect the destination image (Wong et al., 2019; Wiêckowski and Timothy, 2021). Therefore, to comprehend the destination image of a place, one needs first to determine a specific boundary (Chaulagain et al., 2019), since the boundary and the destination image are interrelated. Numerous image studies have focused on country image or city image, which has a clear administrative boundary. However, regional destination image studies are insufficient due to ambiguous spatial boundaries (Park and Song, 2021). Thus, the fluctuating spatial boundary makes it more challenging to measure the regional destination image.

Most previous studies of the regional image regarded a region as one destination and measured the image by one measurement scale (Lee et al., 2012; Kirillova et al., 2020; Park and Song, 2021). This approach could be feasible for regions with resource-based collaboration (Fyall et al., 2012). Since the regional members share similar natural resources (e.g., wine regions), one measurement scale for the whole region is appropriate (Bruwer et al., 2016, 2017; Lefrid and Torres, 2021). However, for some policy-driven regions (Fyall et al., 2012; Park and Song, 2021), the regional members may not demonstrate similarities in terms of natural resources, thus it would be inappropriate to adopt one scale to measure the cognitive image of the region as a whole since the cognitive components of each member are unique. For instance, some places have rich cultural heritage resources while others have more urban settings. If the cognitive image of the region is measured by one scale, the questions may become too broad and general. For example, Kirillova et al. (2020) used 17 items of general descriptions (e.g., clean, accessible, interesting) to measure the cognitive image of the GBA region. However, the unique components of individual regional members were not well presented. As a result, it is difficult to show the complementary advantages of cities in the GBA, that is, the synergy effect. This research attempts to adopt different representative measurement scales for individual regional members. This setting can more accurately evaluate the cognitive image of a region.

### Destination image of the Greater Bay Area

Wiêckowski and Timothy (2021) stated that geographical boundary is an evolving process that involves bordering, de-bordering, and re-bordering. It is especially true for regional development, which usually emerges and goes through this evolving course. Since the opening-up policy was established in 1978, the GBA region has undergone several re-zonings and planning revisions. Xu et al. (2021) stated that the GBA is the former Pearl River Delta (PRD) metropolitan area with the enlargement of Hong Kong and Macau (Park and Song, 2021). The concept of the GBA was proposed in 2016 by integrating the two special administrative regions and nine prefecture-level cities in southern Guangdong Province in the Economic Master Plan. In addition to its rich economic activities, the GBA region is also rich in tourism resources. It features scenic attractions, historical sites, spectacular landscapes, and architecture.

Hong Kong is known as the “modern global metropolis” (Leung et al., 2011; Hsu and Song, 2013; Vo and Nguyen, 2021), and its most famous label should be the “shopping paradise.” According to Huang and Hsu (2005), Hong Kong visitors are primarily motivated by shopping. Leung et al. (2011) concluded that the city is an excellent shopping and sightseeing destination with high accessibility and rich tourism attractions. However, the “shopping paradise” has been losing its price-value advantage and failing to provide a satisfactory service experience in recent years (Law and Cheung, 2010; Jiang et al., 2021). Jiang et al. (2021) suggested that the Hong Kong government needs to diversify tourist attractions and develop new markets. Macau positions itself as a world center for tourism and leisure and a trading platform for Portuguese-speaking countries (Shi et al., 2018). Macau’s cognitive image has been highly correlated with cultural heritage, especially after the historic town was designated as a World Heritage site (Ung and Vong, 2010; Huang et al., 2012). On the other hand, Macau’s destination image is also heavily associated with gambling (McCartney, 2005). The unique image of Macau figures in gambling opportunities, exciting nightlife, luxury hotels, and good infrastructure. Moreover, Macau’s social environment was highly rated by visitors who encountered friendly locals and experienced good service during the trip, contributing to Macau’s positive destination image. The other GBA cities, with Guangzhou as their political and economic hub, provide knowledge and expertise for technological and industrial innovation. Yang and Zhang (2020) argued that Guangzhou is a leisure-oriented city where individuals may “slow down” and feel relaxed in affective perception.

There are increasing regional differences in the GBA region, especially between Hong Kong and mainland China (Kirillova et al., 2020; Wu et al., 2021). In terms of urban formation and identity, these cities differ greatly in size, density, and history (Kirillova et al., 2020). In terms of tourism development, there is currently a lack of a joint tourism governance scheme, which results in an ambiguous and weak destination image of the region. Previous studies of the multi-destination trip have covered areas such as trip patterns (Stewart and Vogt, 1996; Tideswell and Faulkner, 1999; Hwang and Fesenmaier, 2003), travel decision-making (Wu et al., 2012), marketing implications (Hwang and Fesenmaier, 2003), however, there is an apparent lack of study on the multi-destination image of a region. A multi-destination image model should be established to facilitate regional collaboration in tourism.

### Research model and hypothesis

Previous destination image models have demonstrated that cognitive image exerts a great impact on the affective image, which means visitors’ feeling toward a place is largely dependent on the cognitive evaluations and information about that place (Baloglu and McCleary, 1999; Beerli and Martín, 2004; Stylidis et al., 2017). Therefore, the affective image of a regional destination may be influenced by the cognitive image of the region, which is constructed by the member cities. In line with the previous research, the first hypothesis is proposed as follows:

H1: The cognitive image (constructed by different cities) positively influences the affective image of a multi-destination region.

Numerous tourism studies have demonstrated that both cognitive and affective evaluations significantly influence the destination image formation (Huete-Alcocer et al., 2019; López-Sanz et al., 2021) and have a direct effect on the overall image (e.g., Beerli and Martín, 2004; Lin et al., 2007). Therefore, the overall image can be described as an umbrella concept encompassing both cognitive and affective components (Casali et al., 2021). In this study, an overall image of the GBA is the overall impression of the GBA. The following two hypotheses are proposed accordingly:

H2: The cognitive image (constructed by different cities) positively influences the overall image of a multi-destination region.

H3: The affective image of a multi-destination region positively influences the overall image of that multi-destination region.

According to Zhang et al. (2014), both cognitive and affective images affect the conative image positively. Furthermore, based on the numerous studies on the relationship between the overall image and destination components (mainly cognitive and affective images), researchers have argued that the overall destination image not only influences tourists’ choice of destination but also influences their behavior, which is the conative image (Wang and Hsu, 2010; Qu et al., 2011). Therefore, the following hypotheses are developed.

H4: Cognitive image (constructed by different cities) positively influences the conative image of a multi-destination region.

H5: Affective image of a multi-destination region positively influences the conative image of that multi-destination region.

H6: Overall image of a multi-destination region positively influences the conative image of that multi-destination region.

**FIGURE 1:**
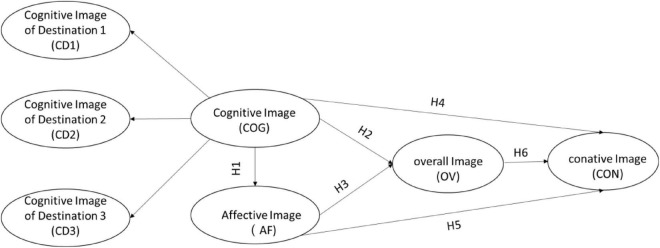
Research framework.

Most of the researchers have employed the same set of items to evaluate the cognitive elements of the destination image of a region (e.g., Kirillova et al., 2020; Park and Song, 2021), but this practice is not suitable for a policy-driven region with multiple distinctive destinations such as the GBA. The novelty of this study is to adopt different representative measurement scales for individual places in the same region. In this way, the cognitive image of the GBA is regarded as a higher-order construct, which is reflected by cognitive images of Hong Kong (Destination 1), Macau (Destination 2), and Guangzhou (Destination 3). Based on the above hypotheses, a research model was established to evaluate how the conative image of a region was influenced by the umbrella term of cognitive, affective, and overall image (Figure 1). The three selected regional members are treated as lower-order constructs in the research model. A confirmatory approach is adopted to assess the proposed framework to explore whether the model is congruent with the data.

## Research methodologies

### Instrument and measures

The questionnaire includes two sections. The first section includes questions that evaluate the constructs proposed in the hypothetical model, while the second section collects demographic information of the respondents. Firstly, the measurement scales of the cognitive image for Hong Kong, Macau, and Guangzhou are retrieved from Choi et al. (1999), Chen et al. (2009), and Lu et al. (2015), respectively. This study did not adopt one scale for the three cities for two reasons. (1) The three cities have distinctive characteristics in terms of cognitive components. Therefore, one set of measurement items could not be applied to all places. (2) Adopting the same set of items to measure the three destination cities may result in common method bias since respondents may rate similar grades for these three cities because they cannot distinguish the differences among the three cities in the same set of measurable items. It was assumed that the scales adopted in the previous research were suitable for this study. If all items of these three scales are entirely borrowed, the number of questions will be 46. A revision was made to shorten the time for taking a survey. Only the items with a factor loading larger than 0.6 in the above sources were used to measure the cognitive image of Hong Kong and Macau. Following Lai and Hitchcock (2017) practice, one measurable item, “Communication is smooth in Hong Kong,” was added to the “communication and language” dimension. Finally, the items for measuring cognitive images were reduced to 38 in total. All items were measured on a seven-point Likert scale.

Four affective image attributes were evaluated using a seven-point semantic differential scale based on previous studies (Baloglu and McCleary, 1999; San Martín and Del Bosque, 2008; Wang and Hsu, 2010). They were distressed-relaxing, unpleasant-pleasant, boring-exciting, and sleepy-arousing. These four items were widely accepted and applied in affective image studies. The overall destination image was measured by a single item asking respondents to which level they agree with the statement, “My overall impression of multi-destination tourism in the GBA is good” (Baloglu and McCleary, 1999; Alcañiz et al., 2009).

As Agapito et al. (2013) suggested, the conative image was captured using three items: “I am likely to take a multi-destination tour in the GBA in the next 2 years,” “I am likely to take a multi-destination tour in the GBA at some point in the future.” and “I am likely to recommend taking a multi-destination tour in the GBA to my friends and relatives.”

### Sampling method and data collection

The target samples are non-Chinese residents who have heard of the concept of the GBA and the cities of Hong Kong, Macau, and Guangzhou. A screening question of “Have you heard of the GBA?” was asked at the beginning of the survey. Then further screening questions of “Have you heard of the city of Hong Kong?”, “Have you heard of the city of Macau?”, and “Have you heard of the city of Guangzhou?” were asked at the start of each sub-section, which evaluates the cognitive image of the three cities in the GBA. A panel of five scholars with solid backgrounds reviewed the questionnaire to ensure content validity. Finally, a face-to-face pilot test was conducted with 50 foreign tourists in Hong Kong, Macau, and Guangzhou to evaluate the content readability of the questionnaire. Several questions in the study have been reworded after the pilot study to improve clarity. The final survey questionnaire items are demonstrated in Table 1.

**TABLE 1:** Measurement items.

3rd order construct	2nd order construct	1st order construct	Item
Cognitive Image of GBA (COG)	CD1: Hong Kong	Activities and atmosphere (HKF1)	HK1 Many interesting places
			HK2 A holiday in Hong Kong is a real adventure
			HK3 Everything is different and fascinating
			HK4 Lots of natural scenic beauty
			HK5 Good quality restaurants and hotels
		Shopping (HKF2)	HK6 Wide variety of products
			HK7 Shopping is convenient
			HK8 Good quality of products
		Culture similarity (HKF3)	HK9 Food is similar
			HK10 Architectural styles are similar
			HK11 Lifestyle and customs are similar
		Communication and language (HKF4)	HK12 Many people speak English
			HK13 Local people are friendly
			HK14 Communication with local is smooth
	CD2: Macau	Culture and heritage (MAF1)	MA1 Macau has interesting cultural and historical attractions
			MA2 The museums and galleries in Macau are interesting to visit
			MA3 World heritage sites are interesting to visit
		Facility (MAF2)	MA4 Macau has good and convention facilities
			MA5 The transportation system in Macau is convenient
			MA6 Tourist information is readily available in Macau
		Urban scenery (MAF3)	MA7 Macau cuisine is unique
			MA8 Macau offers a large variety of events and festivals
			MA9 Macau has attractive climate weathers
		Activity (MAF4)	MA10 There is a variety of nightlife activities in Macau
			MA11 Macau has sufficient sports facilities and activities
			MA12 Macau offers large variety of shopping opportunities
		Comfortability (MAF5)	MA13 It is easy to communicate with people in Macau
			MA14 Macau has attractive natural attractions
			MA15 Macau is easily accessible from my country
	CD3: Guangzhou	Tourism environment (GZF1)	GZ1 Architecture is attractive
			GZ2 Scenery is attractive
			GZ3 Gastronomy is attractive
		Social environment and tourism infrastructure (GZF2)	GZ4 Residents’ friendliness is high
			GZ5 Transportation is good
			GZ6 Service quality is good
		Value and accessibility (GZF3)	GZ7 Price is reasonable
			GZ8 Information is accessible
			GZ9 Crowdedness level is high
		Affective image (AF)	AF1 I think GBA is pleasant
			AF2 I think GBA is relaxing
			AF3 I think GBA is lively
			AF4 I think GBA is exciting
		Overall image (OV)	OV1 My overall impression of GBA is good
		Conative image (CON)	CON1 I am likely to visit GBA in the next 2 years
			CON2 I am likely to visit GBA at some point in the future
			CON3 I am likely to recommend GBA to your friends and relatives

An online survey was conducted from September to October 2021. Convenient sampling was employed in a version of a self-administered questionnaire. A total of 300 questionnaires were collected from five countries (United Kingdom, United States, Japan, New Zealand, and Guinea) located in the five continents in the world (Europe, America, Asia, Oceania, and Africa). In this way, the perceived destination image of the GBA would be more reliable from the perspective of the whole world. Moreover, the selected counties, especially the United States, Japan, and United Kingdom, are important international source markets for China (Statista, 2018). Oceania and Africa markets are also growing and have big potential. For instance, based on the analysis of inbound Chinese tourists with a single stay of more than 4 days, it is found that African tourists comprise the most significant proportion of overnight stays, with Guinea ranked number five in terms of duration of stay (WTA, 2019). Therefore, the selected study sites from the five continents can represent the whole international source markets. After discarding 89 responses with similar grades for most items or with missing data, 211 valid responses were retained. Since the questionnaire is in English, this study selected respondents who can understand English to participate. Filter questions were asked to eliminate those who had never heard of the concept of the GBA (and the three cities–Hong Kong, Macau, and Guangzhou) were selected in this study. Once correctly identified, the respondents were asked to answer the questionnaire according to their experiences.

## Results

In this study, the proposed model was tested by SmartPLS v.3.2.6 due to its suitability for hierarchical models. The PLS method can also be used to small sample size (Wetzels et al., 2009). In addition, PLS allows a non-normal distribution of the data (Goodhue et al., 2012; Hair et al., 2017).

### Sample profile

A total number of 211 valid responses were demonstrated in Table 2. The percentages in gender were similar. 54.5% of the respondents were male, and 45.5% were female. As for the age group, most of the respondents were middle-aged. Only one respondent was below 20 and above 60, respectively. Regarding educational level, more than half of them were university graduates (57.3%). Regarding monthly income level, most respondents were between USD2001-2500 (35.1%) and USD2501-3000 (34.6%). It can be inferred from the above respondent profile that the sample is diversified. Thus, sampling bias can be reduced, and the results can be made more generalizable (Huang et al., 2013).

**TABLE 2:** Profile of respondents.

Demographic characteristics		Frequency	Percentage
Gender	Male	115.0	54.5
	Female	96.0	45.5
Age	18–20	1.0	0.5
	21–30	42.0	19.9
	31–40	82.0	38.9
	41–50	62.0	29.4
	51–60	23.0	10.9
	Above 60	1.0	0.5
Education	Primary school or below	1.0	0.5
	Secondary school/technical	6.0	2.8
	Institution	10.0	4.7
	Tertiary college	36.0	17.1
	University	121.0	57.3
	Graduate student or higher	37.0	17.5
Monthly income (USD)	500 or less	1.0	0.5
	501–1,000	5.0	2.4
	1,001–1,500	3.0	1.4
	1,501–2,000	18.0	8.5
	2,001–2,500	74.0	35.1
	2,501–3,000	73.0	34.6
	3,001 or more	37.0	17.5
Country	Guinea	10.0	4.7
	Japan	43.0	20.4
	New Zealand	18.0	8.5
	United Kingdom	82.0	38.9
	United States	57.0	27.0

### Measurement model

**FIGURE 2:**
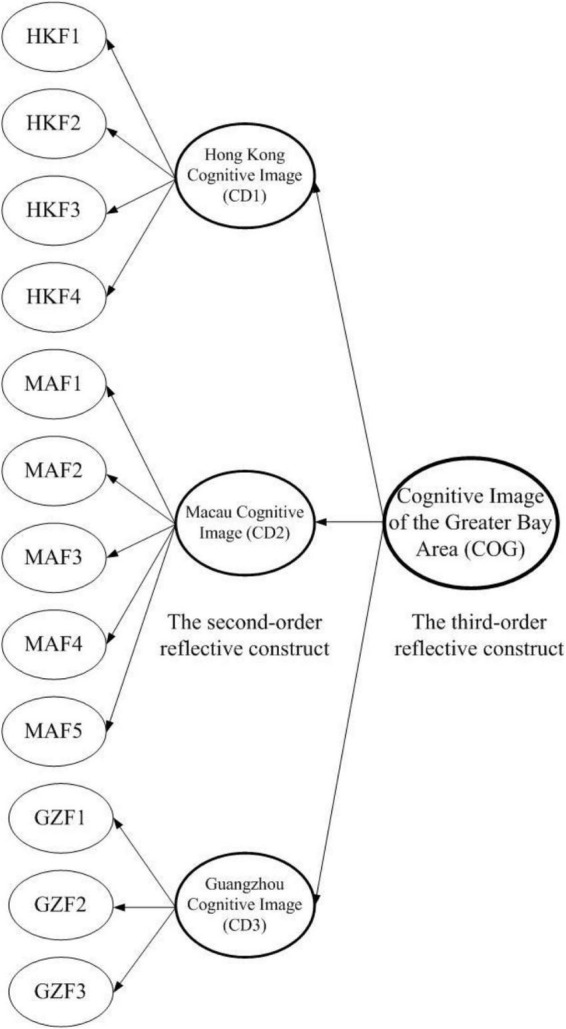
The structure of the overall cognitive image of the GBA.

The factors of the Hong Kong image (HKF1, HKF2, HKF3, and HKF4), Macau image (MAF1, MAF2, MAF3, MAF4, and MAF5), and Guangzhou image (GZF1, GZF2, and GZF3) were the reflective constructs of the first-order constructs, which reflected the cognitive destination image of the three sites (CD1, CD2, and CD3) respectively. CD1, CD2, and CD3 were regarded as the second-order reflective constructs which reflected the third-order construct–the overall cognitive image of the GBA (COG). Figure 2 shows the structure of the formation of the overall cognitive image of the GBA.

The first-order constructs were tested by the Cronbach’s α values, as shown in Table 3. The values for each factor were all higher than the recommended cut-off point of 0.7 (Hair et al., 2010), indicating the scale has good reliability. Composite reliability (CR) measures how accurately each indicator is reflected by the latent construct. In this study, the CR value is above 0.8, and all the average variance extracted (AVE) values of first-order latent constructs compellingly exceed the threshold of 0.5 proposed by Fornell et al. (1981). Discriminate validity was evaluated in both Fornell-Larcker Criterion and the Heterotrait-Monotrait Ratio (HTMT). Table 4 demonstrates that the square root of AVE was larger than the correlation coefficient with the other constructs (Fornell et al., 1981) and all of the HTMT values were less than the threshold of 0.9 (Henseler et al., 2015). Considering statistical significance, the proposed model revealed suitable reliability and validity, indicating that all items were valid.

**TABLE 3:** Reliability and validity for first-order constructs.

3rd order construct	2nd order construct	1st order construct	Item	Outer loading	Cronbach’s alpha	Composite reliability	Average variance extracted (AVE)
COG	CD1	HKF1	HK1	0.788	0.839	0.886	0.610
			HK2	0.791			
			HK3	0.799			
			HK4	0.818			
			HK5	0.703			
		HKF2	HK6	0.814	0.774	0.869	0.688
			HK7	0.846			
			HK8	0.828			
		HKF3	HK9	0.840	0.831	0.899	0.747
			HK10	0.879			
			HK11	0.873			
		HKF4	HK12	0.823	0.773	0.868	0.687
			HK13	0.850			
			HK14	0.813			
	CD2	MAF1	MA1	0.803	0.790	0.877	0.704
			MA2	0.847			
			MA3	0.866			
		MAF2	MA4	0.764	0.710	0.838	0.633
			MA5	0.833			
			MA6	0.789			
		MAF3	MA7	0.811	0.724	0.844	0.644
			MA8	0.793			
			MA9	0.803			
		MAF4	MA10	0.728	0.761	0.863	0.678
			MA11	0.871			
			MA12	0.864			
		MAF5	MA13	0.827	0.756	0.860	0.672
			MA14	0.832			
			MA15	0.800			
	CD3	GZF1	GZ1	0.829	0.791	0.877	0.705
			GZ2	0.830			
			GZ3	0.859			
		GZF2	GZ4	0.808	0.804	0.884	0.719
			GZ5	0.857			
			GZ6	0.877			
		GZF3	GZ7	0.857	0.743	0.806	0.585
			GZ8	0.786			
			GZ9	0.634			
		AF	AF1	0.829	0.842	0.894	0.679
			AF2	0.812			
			AF3	0.828			
			AF4	0.828			
		OV	OV1	1.000	1.000	1.000	1.000
		CON	CON1	0.824	0.807	0.886	0.721
			CON2	0.873			
			CON3	0.850			

**TABLE 4:** Discriminate validity for first-order constructs.

	Fornell-Larcker Criterion														
	AF	CON	GZF1	GZF2	GZF3	HKF1	HKF2	HKF3	HKF4	MAF1	MAF2	MAF3	MAF4	MAF5	OV
AF	***0.833***														
CON	0.695	***0.869***													
GZF1	0.754	0.622	***0.839***												
GZF2	0.738	0.617	0.791	***0.848***											
GZF3	0.761	0.612	0.705	0.758	***0.765***										
HKF1	0.584	0.504	0.641	0.627	0.628	***0.781***									
HKF2	0.385	0.484	0.488	0.519	0.589	0.719	***0.830***								
HKF3	0.563	0.444	0.536	0.547	0.560	0.571	0.561	***0.864***							
HKF4	0.484	0.430	0.537	0.580	0.526	0.671	0.653	0.683	***0.829***						
MAF1	0.495	0.485	0.560	0.596	0.569	0.605	0.556	0.505	0.561	***0.839***					
MAF2	0.558	0.455	0.627	0.660	0.625	0.643	0.485	0.546	0.560	0.727	***0.796***				
MAF3	0.429	0.418	0.582	0.592	0.571	0.612	0.557	0.516	0.615	0.719	0.733	***0.802***			
MAF4	0.547	0.465	0.588	0.597	0.571	0.535	0.433	0.575	0.569	0.690	0.719	0.760	***0.823***		
MAF5	0.556	0.467	0.621	0.623	0.595	0.558	0.490	0.593	0.591	0.680	0.676	0.724	0.745	***0.820***	
OV	0.660	0.595	0.567	0.630	0.617	0.475	0.468	0.375	0.382	0.470	0.517	0.431	0.469	0.513	***1.000***

	**Heterotrait-Monotrait Ratio (HTMT)**														
	
	**AF**	**CON**	**GZF1**	**GZF2**	**GZF3**	**HKF1**	**HKF2**	**HKF3**	**HKF4**	**MAF1**	**MAF2**	**MAF3**	**MAF4**	**MAF5**	**OV**

AF															
CON	0.820														
GZF1	0.816	0.726													
GZF2	0.820	0.733	0.892												
GZF3	0.852	0.840	0.856	0.812											
HKF1	0.670	0.600	0.782	0.763	0.836										
HKF2	0.460	0.590	0.616	0.658	0.851	0.899									
HKF3	0.663	0.535	0.662	0.671	0.757	0.685	0.697								
HKF4	0.542	0.478	0.680	0.732	0.732	0.831	0.843	0.846							
MAF1	0.597	0.593	0.704	0.744	0.796	0.741	0.704	0.624	0.718						
MAF2	0.683	0.567	0.835	0.844	0.811	0.829	0.646	0.711	0.754	0.836					
MAF3	0.523	0.524	0.763	0.773	0.849	0.783	0.742	0.663	0.819	0.849	0.822				
MAF4	0.638	0.569	0.757	0.760	0.814	0.670	0.575	0.718	0.744	0.827	0.832	0.830			
MAF5	0.676	0.561	0.801	0.801	0.856	0.695	0.632	0.748	0.776	0.875	0.832	0.825	0.837		
OV	0.715	0.650	0.633	0.701	0.756	0.516	0.529	0.412	0.431	0.527	0.613	0.505	0.534	0.588	

Bold and italic values indicate the square root of AVE (average variance extracted).

The cognitive destination images of the three cities (CD1, CD2, and CD3) were treated as second-order constructs. The reliability and validity of the second-order constructs were presented in Table 5, and the discriminate validity was presented in Table 6. Following the same approach, the overall cognitive image of GBA (COG) was regarded as a third-order construct. Table 7 shows the reliability and validity results, and Table 8 presents the discriminate validity. The results demonstrate that all high-order constructs have good reliability and validity.

**TABLE 5:** Reliability and validity for second-order constructs.

Construct	Item	Outer loading	Cronbach’s alpha	Composite reliability	Average variance extracted (AVE)
CD1	HKF1	0.883	0.878	0.916	0.732
	HKF2	0.871			
	HKF3	0.803			
	HKF4	0.865			
CD2	MAF1	0.866	0.927	0.945	0.774
	MAF2	0.881			
	MAF3	0.891			
	MAF4	0.895			
	MAF5	0.873			
CD3	GZF1	0.904	0.901	0.938	0.834
	GZF2	0.934			
	GZF3	0.906			

**TABLE 6:** Discriminate validity for second-order constructs.

	Fornell-Larcker Criterion					
	AF	CD1	CD2	CD3	CON	OV
AF	***0.824***					
CD1	0.614	***0.855***				
CD2	0.651	0.735	***0.880***			
CD3	0.810	0.724	0.747	***0.913***		
CON	0.708	0.578	0.555	0.677	***0.849***	
OV	0.702	0.502	0.548	0.663	0.612	***1.000***

	**Heterotrait-Monotrait Ratio (HTMT)**					
	**AF**	**CD1**	**CD2**	**CD3**	**CON**	**OV**

AF						
CD1	0.713					
CD2	0.736	0.818				
CD3	0.831	0.813	0.815			
CON	0.753	0.684	0.640	0.792		
OV	0.765	0.530	0.567	0.697	0.679	

Bold and italic values indicate the square root of AVE (average variance extracted).

**TABLE 7:** Reliability and validity for the third-order construct.

Construct	Item	Outer loadings	Cronbach’s alpha	Composite reliability	Average variance extracted (AVE)
COG	CD1	0.893	0.893	0.933	0.823
	CD2	0.906			
	CD3	0.922			

**TABLE 8:** Discriminate validity for the third-order construct.

	Fornell-Larcker Criterion			
	AF	COG	CON	OV
AF	***0.824***			
COG	0.773	***0.907***		
CON	0.707	0.670	***0.849***	
OV	0.702	0.637	0.611	***1.000***

	**Heterotrait-Monotrait Ratio (HTMT)**			
	**AF**	**COG**	**CON**	**OV**

AF				
COG	0.840			
CON	0.833	0.782		
OV	0.765	0.666	0.679	

Bold and italic values indicate the square root of AVE (average variance extracted).

### Structural model assessment

Bootstrapping with 5,000 subsamples was adopted to test the significance of the relationship between latent variables. Prediction values were demonstrated in Table 9 to test the suitability of explanatory variables. *R*^2^ explains how much change in the endogenous variable can be accounted for by one or more exogenous variables. The *R*^2^-values of the endogenous constructs (affective image, overall image, and conative image) are 0.597, 0.515, and 0.553, which are all above 0.5, indicating that the model has a relatively high predictive power (Hair et al., 2010). *f*^2^-values were calculated to investigate whether the omitted structure impacts endogenous variables (Hair et al., 2017). The predictive effect index (*f*^2^) of the cognitive image on the affective image is 1.484, which suggests a very big predictive effect. *f*^2^ of the cognitive image and affective image on the overall image are 0.045 and 0.226, indicating small to medium effects. *f*^2^ of the cognitive, affective, and overall images on the conative image are 0.061, 0.104, and 0.034, suggesting small predictive effects. A model is predictively relevant if *Q*^2^ > 0, while *Q*^2^ < 0 represents a lack of predictive relevance (Vinzi et al., 2010). The *Q*^2^-values are between 0.392 and 0.504, which reveal a large predictive relevance (Henseler et al., 2009).

**TABLE 9:** The results of the prediction values.

	*R* ^2^	*f* ^2^	*Q* ^2^
H1: COG → AF	0.597	1.484	0.402
H2: COG → OV	0.515	0.045	0.504
H3: AF → OV		0.226	
H4: COG → CON	0.553	0.061	0.392
H5: AF → CON		0.104	
H6: OV → CON		0.034	

**FIGURE 3:**
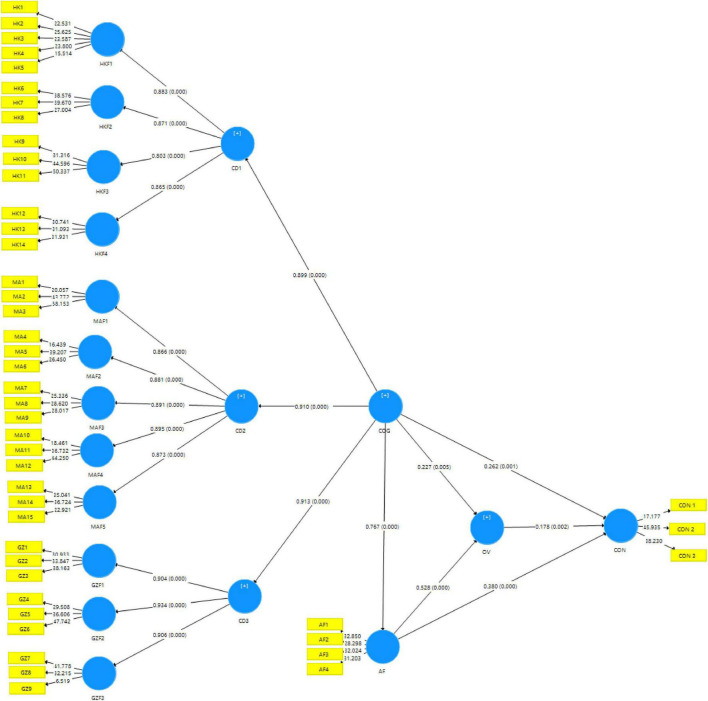
Results of PLS-SEM analysis of model.

The results are presented in Table 10 and Figure 3. The findings suggest that in the GBA region, the cognitive image (COG) has a significant positive effect on affective image (AF) (β = 0.767, *t* = 20.895, *p* < 0.001), overall image (OV) (β = 0.227, *t* = 2.787, *p* = 0.05), and conative image (CON) (β = 0.262, *t* = 3.247, *p* = 0.001). The affective image significantly influences overall image (β = 0.528, *t* = 6.507, *p* < 0.001) and conative image (β = 0.380, *t* = 4.856, *p* < 0.001). The overall image also has a significant but slight effect on the conative image (β = 0.178, *t* = 3.048, *p* = 0.002). Therefore, all the hypotheses (H1–H6) are supported.

**TABLE 10:** Path coefficient and hypothesis testing.

	Path coefficients	Standard deviation (STDEV)	T statistics (| O/STDEV|)	*P*-values	Support
H1: COG → AF	0.767	0.037	20.895	0.000	Yes
H2: COG → OV	0.227	0.081	2.787	0.005	Yes
H3: AF → OV	0.528	0.081	6.507	0.000	Yes
H4: COG → CON	0.262	0.081	3.247	0.001	Yes
H5: AF → CON	0.380	0.078	4.856	0.000	Yes
H6: OV → CON	0.178	0.059	3.048	0.002	Yes

## Discussion and conclusion

The results of data analysis indicate that cognitive images of three cities in the GBA take different weighting in constructing the overall cognitive image of the GBA. This overall cognitive image of the GBA develops the affective image, overall image, and conative image of the GBA. For constructing the conative image, the affective image shows the greatest impact, overall cognitive image follows; the impact of the overall image is less. These results contradict the study by Agapito et al. (2013). They found that the effect of cognitive image on a conative image is stronger than the effect of the affective image of a tourist destination. Agapito et al. (2013) did not consider the effect of the overall destination image. This is because, in this study, the overall cognitive image of the GBA is composed of three cities, which is relatively indistinct compared to a single tourist destination image. Therefore, compared with the affective image of the GBA, the strength of potential tourists’ overall cognitive image of the GBA is relatively weak.

### Theoretical implications

This study contributes in various ways to understanding a regional destination’s image. Firstly, this study is an empirical study to measure the regional destination image separately from the perspective of its regional members, which provides a new angle of measuring regional destination image. Most destination image studies considered a region or country as one single destination. Few empirical studies have examined the cognitive perception of a region and country in the context of spatial differences and different measurement scales of members of the region or country. Park and Song (2021) stated that an image of a regional destination is not simply a synthesis of the images of its member cities or dominated by the image of one popular destination of a region. Therefore, it is inappropriate to use a single measurement scale to measure the cognitive image of all members of the region or country. In this study, different scales of cognitive evaluation were applied to Hong Kong, Macau, and Guangzhou, the three representative cities in the GBA. This approach enhances the accuracy of cognition evaluation and allows comparison effects of the cognitive image among regional members. As shown in Figure 1, the entire research framework can provide researchers with better use of multi-destination tourism research.

Secondly, this study contributes to regional tourism research by exhibiting the evolving process of spatial bordering. If the overall destination image is weak, can the GBA tourism strategy not achieve the expected goals? Or it still supports that the tourism attributes of the cities in the GBA have complementary functions. This study also shows that the cognitive image constructed by three cities strongly affects the affective image of GBA tourism (β = 0.767), and the affective image also strongly affects the conative image (β = 0.380). It implies that tourists are more familiar with individual cities, thereby enhancing the affective image of the GBA as a multi-destination. Therefore, there is a complementary effect. This study helps researchers understand the mechanism of building the conative image of a multi-destination (COG → AF → CON). This mechanism is a de-bordering and re-bordering mechanism of spatial boundaries. The building of affective image of GBA multi-destination is a key to turning tourists’ perception from individual cognitive images of cities (de-bordering) to a whole conative image of a region (re-bordering).

Finally, this study proposes a suitable research method for regional destination image study. It is the first study that considers a region’s image as a higher-order construct which is reflected by individual regional members’ images as lower-order constructs. As shown in Figure 2, the reflective-reflective higher-order construct is deemed appropriate in the destination image study of the multi-destination region. Since measurement of a region should not be based on a single scale, a higher-order construct could be applied to include multiple latent variables, which represent cognitions of different places. Moreover, it is unnecessary to include all the places within a region since the reflective-reflective construct enables the whole region’s image to be reflected by certain representative members. Moreover, for each destination member, suitable evaluative dimensions could be developed accordingly as a lower-order construct. Researchers can apply this multi-destination image model to future regional studies. Most importantly, this study contributes to destination image research by demonstrating a method (the construction of high-order cognitive image structure) for evaluating the multi-cognitive images on other constructs.

Overall, this study fills the research gap in tourism image study in the multi-destination region by evaluating individual regional members’ images and examining the impact of each member’s image on the cognitive evaluations of the overall region. Furthermore, the higher-order construct is applied for a multi-destination image model, contributing to the research methodology on image study.

### Practical implications

This study offers practical implications for several parties, including governments, tourism marketers, and travel agencies. First, the results of this study provide guidelines for tourism destination governments and marketers. The zoning of Guangdong province has changed from the PRD to the Pan-PRD and then to the GBA region over the years. The process of continuously re-bordering raises the issue of re-branding. To re-brand a region, destination management organizations should define their region based on each member’s unique resources. The cognitive image of the GBA is reflected by all the member cities, not dominated by just one member. Understanding how a regional image is formed could help destination marketers to develop a suitable image (Assaker, 2014). The results also demonstrate that the impact of the overall image of the GBA on tourist behavior is rather weak. At the current stage, it may imply that tourists recognize Hong Kong, Macau, and Guangzhou as independent destinations rather than a well-integrated region. This finding echoes with previous scholars that there is a need to establish a destination management organization for the whole GBA region (Kirillova et al., 2020). In light of the growing competitive nature of tourism destinations, collaborative strategies, such as co-branding within a region, are frequently applied to enhance destinations’ market positioning (García et al., 2012). Co-operative branding is proven effective for regional tourism development, which could help a region build a stronger image and enhance its identity (Cai, 2002). For policy-driven regions like the GBA, which may not share similar resources, wise integration of resources could bring cost-effectiveness. The region members could complement each other and perform better as a whole than individuals. Collaborating among destinations is also beneficial from the perspective of strategic place branding since it increases competitiveness to a sustainable level while signaling the unique qualities of offered products, services, and experiences (Haugland et al., 2011; Kirillova et al., 2020). Moreover, according to the study results, the smallest reflective loading of the Hong Kong image (0.893) indicates that the Hong Kong cognitive image is distinctive from the (overall) cognitive image of GBA. The results imply that international tourists perceive a more independent cognitive image of Hong Kong. Since the GBA includes both mainland cities and two special administrative regions, the government needs to bridge cross-border gaps in political and cultural views to achieve better integration.

Second, this study provides recommendations for travel agencies. Few package tours currently include multiple destinations when searching for the GBA on popular Chinese travel agency websites. Foreign tourists have even fewer products to choose from. Thus, travel agencies are suggested to design tourism products that combine each city’s distinctive features and complementary resources to maximize income. To make the GBA region a top tourist destination in the world, we must consider the perspective of foreign markets. Therefore, comprehensive tour products which include multiple cities in the GBA should be developed and promoted. The study result implies a lack of integrated image for the GBA region. Thus, travel agencies should provide more online information about GBA tour products, local culture, facilities, and visa application process to increase the awareness of the GBA for global tourists. Moreover, one trip to multiple destinations is not limited to the GBA. Many countries and regions worldwide have also implemented similar tourism concepts for multi-destination regions, especially for long-distance travelers. As the COVID-19 pandemic will be under control with coordination among countries and sustainable development, international tourism is predicted to revive again soon (Abbas et al., 2021; Marek, 2021).

## Limitations and future research

There are several limitations to this study. First, this study did not focus on scale development for different destination cities in the GBA. Instead, it was assumed that the adopted scales from previous research are suitable to measure the cognitive components of Hong Kong, Macau, and Guangzhou. In future studies, researchers could develop updated measurement scales of each regional member by using interviews with tourists or online comments from tourists. Second, this study only included three leading members in the GBA region. Since the GBA is also an emerging concept, other members in Guangdong province could be studied in the future. Third, this study targeted overseas travelers to the GBA. Due to the relatively small sample size, this study did not compare the demographic characteristics of visitors from different countries. Future research could specify demographic differences to provide a basis for selecting a target market and designing suitable market campaigns for a different market. Finally, this study provided a framework to study the multi-destination image. Subsequent research can compare the influence of various regional members on the destination image of a region so that strategic recommendations can be put forward for destination branding of the whole region.

## Data availability statement

The raw data supporting the conclusions of this article will be made available by the authors, without undue reservation.

## Ethics statement

Ethical review and approval was not required for the study on human participants in accordance with the local legislation and institutional requirements. Written informed consent from the participants to participate in this study was not required in accordance with the national legislation and the institutional requirements.

## Author contributions

XD collected the data, performed data analysis, drafted the manuscript, and revised the manuscript. IL directed the manuscript writing, revised the manuscript, and managed the manuscript submission. Both authors contributed to the development of the research framework and contributed to the article and approved the submitted version.
