# Corrigendum: The formation of multi-destination image: A study of China's Greater Bay Area

**DOI:** 10.3389/fpsyg.2022.1083458

**Published:** 2022-11-15

**Authors:** Xialei Duan, Ivan Ka Wai Lai

**Affiliations:** Faculty of International Tourism and Management, City University of Macau, Macao, Macao SAR, China

**Keywords:** destination image, structural equation modeling, higher-order construct, regional tourism, Greater Bay Area

In the published article, there was an error in the **Funding** statement as published. The wrong grant was listed. The correct **Funding** statement appears below.

## Funding

This study was supported by the Education Fund of the Macao SAR Government under the Specialized Subsidy Scheme for the Tourism Education and Training for the Guangdong-Hong Kong-Macao Greater Bay Area (TET-CITYU-2020-02).

The authors apologize for this error and state that this does not change the scientific conclusions of the article in any way. The original article has been updated.

## Publisher's note

All claims expressed in this article are solely those of the authors and do not necessarily represent those of their affiliated organizations, or those of the publisher, the editors and the reviewers. Any product that may be evaluated in this article, or claim that may be made by its manufacturer, is not guaranteed or endorsed by the publisher.

